# Spatial heterogeneity of gut microbiota reveals multiple bacterial communities with distinct characteristics

**DOI:** 10.1038/srep06185

**Published:** 2014-08-26

**Authors:** Hsiao-Pei Lu, Yung-Chih Lai, Shiao-Wei Huang, Huang-Chi Chen, Chih-hao Hsieh, Hon-Tsen Yu

**Affiliations:** 1 Department of Life Science, National Taiwan University, Taipei, Taiwan, ROC 10617; 2 Institute of Oceanography, National Taiwan University, Taipei, Taiwan, ROC 10617; 3 Institute of Ecology and Evolutionary Biology, National Taiwan University, Taipei, Taiwan, ROC 10617; 4 Genome and Systems Biology Degree Program, National Taiwan University, Taipei, Taiwan, ROC 10617; 5 Current address: Institute of Oceanography, National Taiwan University, Taipei, Taiwan, ROC 10617.

## Abstract

We analyzed bacterial communities of six distinct gut sites (the food bolus and mucus layer of the proximal small intestine, cecum and distal large intestine), using wild folivorous flying squirrels. We found significant spatial heterogeneity in composition, diversity, and species abundance distributions (SADs) of gut microbiota, corresponding to physicochemical conditions. High diversity was detected in the mucus layer of small intestine and the food bolus of cecum, followed by the food bolus of large intestine and the mucus layer of cecum, and relatively low diversity in the food bolus of small intestine and the mucus layer of large intestine, likely due to disturbance and resource partitioning. The SADs showed succession-like patterns in the food bolus communities from the proximal to distal gut. Notably, each mucus layer community had a unique pattern different from the food bolus community of the same compartment, with distinct relative abundances of dominant species. In combination with data from other mammalian fecal samples, we concluded that gut microbiota were apparently dynamic in community structure, from low species richness with unequal abundances to high species richness with equal abundances; these findings were interpreted as strong habitat effects on bacterial communities.

The mammalian gut is a complicated ecosystem with several compartments (from the mouth to anus) involving a series of steps (mechanical, chemical, microbial) in breaking down foods, with microbiota having an important role^1^,^2^. Gut microbiota living in resource-rich environments have co-evolved with mammals for millennia, forming a close symbiotic relationship^3^. Food boluses in various compartments along the longitudinal axis of the gut might be colonized by distinct microbiota, corresponding to various physicochemical conditions^1^,^2^. Moreover, in the radial axis of the same compartment, the gut mucus layer composed of host-secreted sugars^4^,^5^ might provide microbiota a habitat that is distinct from the food bolus. Several authors have suggested that bacterial communities from various gut sites might be of distinct composition and function^6^,^7^,^8^,^9^,^10^, whereas most studies only used fecal samples to represent gut microbiota. To understand the complexity of the gut ecosystem, herein we investigated spatial heterogeneity of intestinal bacterial communities, including differences among gut compartments (along a longitudinal axis) and differences between the food bolus and mucus layer (along a radial axis).

In the present study, wild folivorous flying squirrels (*Petaurista alborufus lena*) were used to understand the spatial pattern of gut microbiota in small herbivores. Gut microbiota of the latter are of interest because they are expected to function as an efficient digestive system^11^, helping the host maintain a relatively high metabolic rate compared to large herbivores^1^,^2^. In contrast to the enlarged forestomach of ruminants, small herbivores typically have an enlarged cecum for microbial breakdown of plant fibers^1^,^2^. Indeed, the cecum of the flying squirrel has been shown to be functionally similar to the bovine rumen, containing diverse microbiota for food processing^12^. To investigate spatial heterogeneity of gut microbiota, bacterial communities of six gut sites (the food bolus and mucus layer of the proximal small intestine, cecum and distal large intestine) were investigated using a culture-independent 16S rRNA gene survey^13^. Further, community features of various gut sites were characterized and compared in several aspects, including species composition, species diversity and species abundance distributions (SADs) with taxonomic information.

The species composition of microbiota in the mammalian gut ecosystem is highly variable^3^. As previously reported within and between mammalian hosts, >50% of species are unique in a fecal sample, with no single species identified in all samples^14^,^15^. Therefore, instead of exploring details regarding species identity, in this study, species diversity^16^ and SADs^17^ were applied to characterize spatial patterns of gut microbiota. These two approaches facilitate comparison of very dissimilar communities (including those without species in common)^17^. More importantly, as species diversity and SADs have been intensively studied in macro-organisms (animals and plants), here we adopted their deriving rules^16^,^17^ to predict spatial patterns of intestinal bacterial communities.

Species diversity incorporates two components of community structure, namely richness (the number of species) and evenness (consistency of abundances among species), representing fundamental features of biological assemblages^16^. To make predictions on species diversity among gut sites, we considered two ecological factors, namely productivity (resource quality) and stability (disturbance intensity), known to strongly influence species diversity of macro-organisms in many ecosystems^18^,^19^,^20^. According to physicochemical conditions (pH and host secretions) of the mammalian gut^21^,^22^, we formulated two hypotheses: 1) For the bacterial community infiltrating the food bolus, we hypothesize that there is low species diversity in the small intestine (where rapid flow of digestive fluids as strong disturbance)^23^,^24^, but much greater species diversity in the cecum and large intestine (where relatively stable environments for microbes to utilize dietary resources)^1^,^2^; 2) For the bacterial community embedded in the mucus layer, we hypothesize that host-secreted sugars^4^,^5^ as simple enriched nutrients^25^,^26^ would result in low species diversity of this habitat, compared to complex substrates in the food bolus.

In addition to species diversity, SADs provide information regarding how species partition resources (the relative abundance of a species is proportional to the amount of resources it occupies)^27^,^28^, offering further insights into community organization. Furthermore, it has been proposed that each bacterial phylum has its own biological properties and forms a unique ecological coherence^29^; therefore, in the present study, we integrated phylum-level taxonomic information into traditional SADs^17^ to advance our understanding regarding spatial distributions of disparate bacterial phyla in the gut ecosystem. These SADs would enable characterization of relative abundances of dominant/rare species and determination of whether or not they belong to the same phylum. Moreover, as explained by the niche hierarchy model^27^,^28^, we expected that the SADs of the food bolus communities (portioning heterogeneous resources) would be more equitable than those of the mucus layer communities (portioning homogeneous resources). In addition, we expected a highly uneven SAD in the food bolus of the small intestine, since the community there was just reconstructing after exposure to harsh chemicals (gastric acid and bile)^24^, whereas communities of the cecum and large intestine would represent even SADs as they were relatively isolated from disturbance.

In the present study, spatial patterns of intestinal bacterial communities in small herbivores were characterized by investigating species composition and diversity, as well as SADs annotated with phylum-level taxonomy. Perhaps the “wild” flying squirrel used here would reflect community structure of gut microbiota under natural environmental conditions with long-term evolutionary effects. More importantly, our objective was to provide an ecological interpretation regarding observed patterns, based on community assembly rules derived from macro-organisms. We expected that concepts of species diversity and SADs could be applied to not only plant and animal communities, but also to bacterial communities, even though the ecological definition of species may differ in specific cases.

## Results

### Gut microbiota exhibited substantial spatial heterogeneity in species composition

**Figure 1:**
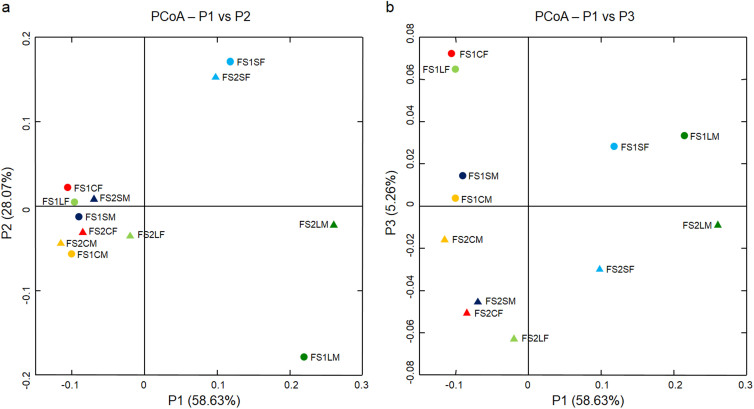
Biplots showing relationships of 12 intestinal bacterial communities. The scores for P1 and P2 (the first and second axes) are plotted in (a) and the scores for P1 and P3 are plotted in (b) using principal coordinates analysis (PCoA) based on the UniFrac distance. For each flying squirrel (FS1 or FS2), samples were prepared from the proximal small intestine (S), cecum (C) and distal large intestine (L), with each section divided into the food bolus (F) and mucus layer (M). Symbols (circle and triangle) represent two individual hosts and six distinct colors indicate different gut compartments. The SF and LM were distinguished from the other communities by P1, whereas communities from the two individuals were separated by P3.

**Figure 2:**
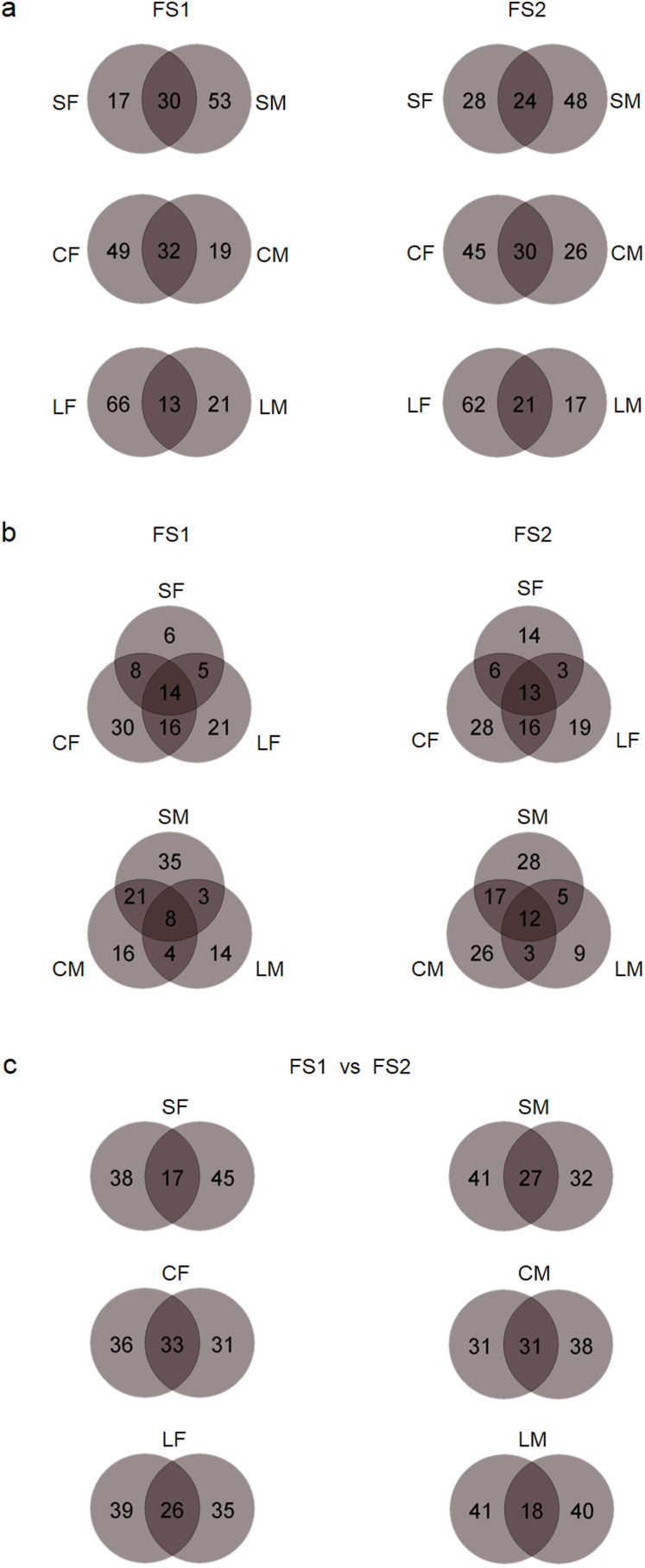
Species overlap patterns between or among the intestinal bacterial communities. Patterns of gut sites within one individual (a and b) and patterns of the same gut site from the two individuals (c) are shown with the percentage (%) of overlapping species (community labels were defined in Fig. 1).

Considerable spatial heterogeneity of bacterial species composition was found among gut sites (Fig. 1). Based on ordination analysis (incorporating both species abundance and phylogenetic information), gut habitat effects (resource quantity and quality) strongly affected species composition of the intestinal bacterial communities (Fig. 1a). The SF (food bolus of the small intestine) and LM (mucus layer of the large intestine) were clearly separated from the remaining communities in the first axis (Fig. 1a), implying that the environmental conditions of the SF and LM were distinctively different from the rest of the gut. In addition, communities from the two individuals were roughly separated in the third axis (Fig. 1b), suggesting that host individual effects (animal genetic and physiological conditions) also had influences on species composition. To reveal influences of gut habitats and host individuals on bacterial communities, we checked overlap patterns considering only the presence/absence of species (Fig. 2). There was high variation in species members among those communities, with limited overlap, not only between the food bolus and mucus layer (Fig. 2a), but also among food boluses or mucus layers across all three gut compartments (Fig. 2b). Moreover, the species members of the same gut site also varied considerably between the two individuals, with only ~20 to 30% shared (Fig. 2c). Taken all together, we concluded that gut bacterial communities were structured by effects from both local habitat conditions and host individual differences, of which local habitat effects determined the main community structure (which phylogenetic groups could become abundant in which habitats) and host individual effects determined the detailed composition of species members.

It is noteworthy that, the LF (the food bolus of the large intestine) in this study could be regarded as an end product of food degradation (similar to fecal samples). Based on the results, the bacterial community in the LF was most similar to that in the CF, but distinct from those of the SF and LM (Figs. 1 and 2). Therefore, our findings reinforced previous observations^6^,^7^,^8^,^9^,^10^ that using a fecal sample to represent an overview of gut microbiota would very likely fail to detect community variation responding to physicochemical conditions of the gut, especially for the upper gut or mucus-associated communities.

### Species diversity (both richness and evenness) varied drastically among gut sites

Strong spatial heterogeneity of bacterial species composition was consistent with substantial differences in environmental conditions among gut sites^1^,^2^. In addition to the composition, diversity also varied drastically among gut sites (Table 1). The mucus layer of the small intestine (SM) and the food bolus of the cecum (CF) had relatively high species diversity (estimated richness and two Shannon's indices), followed by the food bolus of the large intestine (LF) and the mucus layer of the cecum (CM), whereas the food bolus of the small intestine (SF) and the mucus layer of the large intestine (LM) had relatively low species diversity (Table 1). These spatial differences of diversity were confirmed by rarefaction curves of both observed species richness and the Chao1 estimated richness after controlling sampling efforts (Supplementary Fig. S1). Furthermore, the initial slopes of the rarefaction curves, which reflect the probability that two randomly picked sequences belong to different species^30^, showed similar variation as evenness (Table 1). Accordingly, differences in species diversity among gut sites were detected by distinct diversity measures, of which two components of species diversity (richness and evenness) revealed compatible patterns: communities in the SM and CF were diverse and even, whereas those in the SF and LM were simplified and uneven in community structure.

### Different bacterial phyla were not uniformly distributed among communities

**Figure 3:**
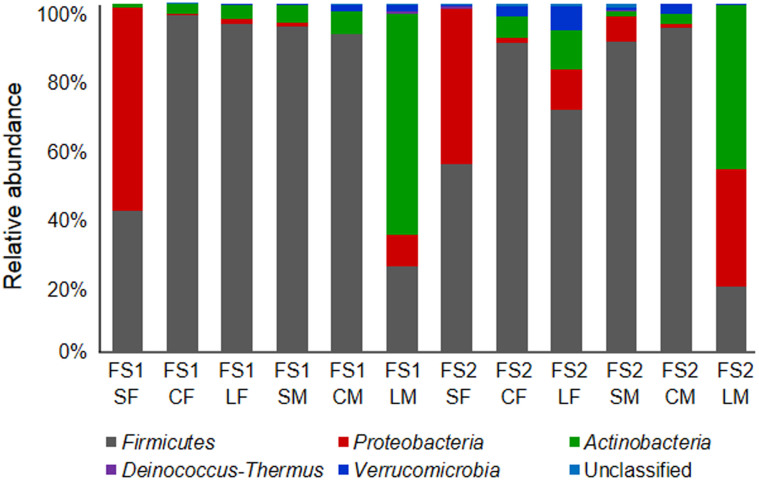
Taxonomic composition (phylum level) of the 12 intestinal bacterial communities. The 16S rRNA sequences from all communities were classified into five bacterial phyla, with <1% unclassified bacterial sequences (community labels were defined in Fig. 1).

Phylum composition of bacterial communities from distinct gut sites varied from each other, with the SF and LM communities showing substantial differences from the other communities (Fig. 3). *Firmicutes* were prevalent in all communities, whereas a large proportion of *Proteobacteria* and *Actinobacteria* were detected in the SF and LM communities, respectively (Fig. 3). Since the SF and LM communities had apparent lower species diversity than the other communities (Table 1), different phyla were expected to contain high to low levels of species richness (as well as various abundances per species) across gut sites. Consistent with the expectation, when phylum information was assigned to each species, distinct richness/abundance patterns were detected among various bacterial phyla (Table 2). Phylum *Firmicutes* had the highest species richness in all gut sites, with low mean species abundance ranging from 1 to 3 (most *Firmicutes* species were rare species in all communities). In contrast, *Proteobacteria* and *Actinobacteria* contained much fewer numbers of species, with relatively high mean abundance in some gut sites (they were often dominant species when they occurred). Moreover, each of *Deinococcus-Thermus* and *Verrucomicrobia* was represented by only a single species with high variation in abundances across communities (Table 2). Therefore, we inferred that species of different phyla might vary markedly in habitat preferences and/or functional responses to gut environments, consistent with the ecological coherence of high bacterial taxa^29^.

### Species abundance distributions displayed dynamic community structural changes

**Figure 4:**
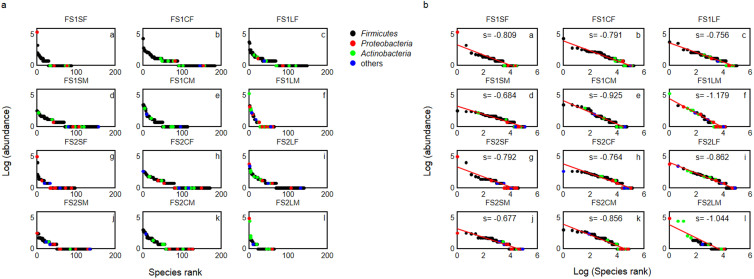
Species abundance distributions at a rank-log space (a) and at a log-log space (b). Species are ranked in order from the most abundant to the least abundant for each intestinal bacterial community. Each dot represents a species and is colored according to its phylum (*Firmicutes*, *Proteobacteria*, *Actinobacteria*, and others; the latter includes *Deinococcus-Thermus*, *Verrucomicrobia*, and unclassified bacteria). The red line in (b) represents the best power-law fit, with the power-law exponent (s) shown in each panel (community labels were defined in Fig. 1).

Species abundance distributions (SADs) provided complementary insights regarding habitat effects on bacterial community structure. First, rank-abundance curves (Fig. 4a) of the SF (Panels a and g) and LM (Panels f and l) communities decayed faster than all the other communities, whereas the curves of the other communities declined gradually with a relatively longer “tail” (more rare species). In addition, in log-log plots (Fig. 4b), the top few species of the SF (Panels a and g) and LM (panels f and l) communities deviated significantly upward from the power-law fitting line, representing higher-than-expected abundances. Specifically, the top rank species of the SF and LM communities occupied ~30 to 60% of total abundances, whereas the most dominant species of the other communities accounted for only ~3 to 15% of total abundances. Whilst the power-law approach assumes that there is an equilibrium in the community, this does not require that the equilibrium is always achieved. For example, considering disturbance that occur on time scales faster than those for reaching the equilibrium, we would detect some species with high abundances as they may benefit disproportionately from disturbance relative to others. Combining the SAD patterns with results of species diversity (Table 1 and 2), we concluded that the SF and LM communities might be under unbalanced states with a few well-adapted *Proteobacteria* and *Actinobacteria* species taking a big portion of the resources, whereas the other communities were characterized by diverse *Firmicutes* species which evenly shared resources^27^,^28^.

## Discussion

Our findings demonstrate clear spatial heterogeneity of bacterial communities in the gut ecosystem of small herbivores. These differences can be interpreted as habitat effects associated with gut compartmentation. For example, along the longitudinal axis, as local disturbance usually induces loss of species^31^, host digestive fluids (gastric acid and bile) might be the determining factor in the proximal part of the gut, resulting in low diversity in the SF communities. By contrast, as the food bolus moved away from the source of disturbance, diversity increased from the proximal to distal parts of the gut, with high diversity in the CF and LF communities (Table 1). This pattern is similar to that involving macro-organisms, as shown in species recovery patterns after storms, floods, or fires^23^. In addition to diversity, the structural changes of the food bolus communities from the small to large intestine (Panels a–c and g–i of Fig. 4) had succession-like patterns consistent with classic findings in plant communities^25^,^26^. The SF communities had SADs similar to plants in the early stage of succession, whereas the SADs of the CF and LF communities were more like those in later stages of succession^25^,^26^. Likewise, the dominant species (*Proteobacteria* species) of the SF may have had a competitive advantage (such as rapid growth) in unstable gut environments (similar to “weed” species), whereas most species (*Firmicutes* species) of the CF and LF may mutualistically interact with each other to form a tight “commonwealth” facilitating synergistic food degradation.

Beyond indicating high diversity in the distal gut, we emphasized that the number of bacterial species was greater in the cecum than in the large intestine (CF > LF; Table 1). As each species may contribute a distinct set of enzymes and diverse members would result in intricate interactions for food processing^32^, high species diversity in the cecum implies that the spatial pattern of bacterial diversity may vary according to functional specialization of the digestive system, with the highest species diversity in the main food-processing gut compartment^12^. As higher microbial diversity in soil and sludge can hasten the rate of decomposition of organic and xenobiotic compounds^33^,^34^, higher bacterial diversity in the flying squirrel's cecum may facilitate efficient energy extraction from the recalcitrant leaf-based diet. Moreover, analogous with patterns in macro-organisms^35^,^36^, species-rich cecal communities may be more stable in biomass production and recover more rapidly from disturbance. Further studies focusing on relationships between species diversity and functional diversity^37^ should provide insights regarding the impact of bacterial community structure on the gut ecosystem functioning.

Regarding the radial axis in the small intestine, the mucus layer (SM) unexpectedly^38^,^39^ had much higher bacterial species diversity than the food bolus (SM > SF; Table 1). It was noteworthy that the diversity of the SM was as high as the CF (the food bolus of the cecum). The SM was expected to have low diversity, as this area has numerous antimicrobial molecules and generally contains fewer microbial cells^40^. However, we detected the SM with the highest diversity among gut sites, suggesting that this area may serve as a reservoir of diverse bacterial species. A healthy mucus layer with various immune factors has long been recognized as a barrier to preventing infection by vast numbers of microbes^40^, whereas there is increasing evidence that the mucus layer as the primary site for the host-microbial interactions might play critical roles in licensing and managing the symbiotic microbiota through well-regulated molecular processes^41^,^42^. Accordingly, based on the current findings, we inferred that diverse bacterial species may be selected or tolerated in the mucus layer of the small intestine, and thereafter colonize the cecum (in numerous cell numbers) for food degradation. In that regard, SM and CF shared approximately one-third of the species members (data not shown), more than between SM and SF (Fig. 2a). Further studies are awaited to verify our observations and to investigate how diverse bacterial species are maintained in the SM.

By contrast to the radial discrepancy of the small intestine, mucus layers of the cecum and large intestine (CM and LM) had less bacterial species diversity than food boluses of the same compartments (CF and LF). In addition to diversity, CM and LM had a stronger niche partitioning gradient (steeper slopes in Fig. 4b) than CF and LF, confirming the notion that the mucus layer (which is full of host-secreted sugars)^4^,^5^ may represent a more homogeneous resource base^27^,^28^ than the food bolus. Moreover, the lowest diversity detected in the LM may be explained by biochemical characteristics of the mucus layer^4^,^5^. In the murine and human gut, thickness and acidity of the mucus layer varied along the length of the gut, with a relatively thick and acidic glycan layer in the large intestine^4^,^5^. This glycan layer providing extra carbohydrate sources might enhance fitness of certain bacteria^43^,^44^. Thus, although the LM might contain numerous bacterial cells^38^,^39^, most of them belong to a few well-adapted species (*Actinobacteria* and *Proteobacteria* species; Fig. 4 and Supplementary Fig. S2), which probably were able to adhere to the mucus layer and used host-secreted sugars as energy sources.

Our interpretation about community structure was based on the consistent patterns of two flying squirrel individuals, which may not be enough to robustly demonstrate how intestinal bacterial communities change in response to local environmental conditions. Nevertheless, this study (and our unpublished results with more individuals in other animals) pointed out the importance of investigations into multiple gut sites. Future research focusing on more individuals from more kinds of animals is demanded to capture overall spatial heterogeneity of the gut microbiota, considering intro-host and inter-host variation in gut structure. For example, we may investigate what structural similarities/differences between rumen microbiota of large herbivores and cecal microbiota of small herbivores.

In a general theory of community ecology^45^, relative abundance patterns are related to the maintenance of species diversity; that is, rare species are numerically closer to extinction in communities with uneven abundance patterns (the abundance ratio of dominant to rare species is high) than in those with even abundance patterns. Accordingly, when the gut environment is simplified by disturbance or enriched nutrients (situations in the SF and LM), the existence of rare species would potentially be threatened by those extremely dominant species which monopolize most resources in the habitats.

To reveal how community structure changed with the shift of species abundances, we further investigated relationships among distinct diversity measures (richness, diversity, evenness, and top rank species dominance) using correlation analysis. Both richness and evenness were positively correlated with diversity (r > 0.93, P < 0.01; Supplementary Fig. S3a) across the 12 intestinal bacterial communities. Conversely, both richness and evenness had negative correlations with the dominance of the top rank species (r < −0.78, P < 0.01; Supplementary Fig. S3b). To generalize community structure dynamics, species diversity patterns of two published data sets^14^,^15^ containing sufficient bacterial communities from mammalian fecal samples were examined. Species richness, diversity, and evenness were highly interrelated across communities (Supplementary Fig. S4a and S5a). Importantly, the dominance of the top rank species indeed had strong negative influence on both richness and evenness (Supplementary Fig. S4b and S5b). Although our results demonstrated that richness and evenness generally increased or decreased in parallel in gut bacterial communities, there is still a need for more comprehensive analyses in other microbial communities, because inconsistent patterns have been reported in previous studies on macro-organisms^46^,^47^. For example, correlations between richness and evenness were typically positive in animal communities and negative in plants and fungi^47^, but no significant relationship was detected in some cases^46^.

Community diversity may have an important impact on ecosystem functioning^48^,^49^,^50^. Although species richness is the most intuitive measure of community diversity^16^, species evenness considering relative abundances of species members should be regarded as more reliable and suitable for studying gut microbiota, because the latter is more robust^51^ and often more sensitive to environmental changes^47^. Furthermore, despite substantial evidence that losing species would strongly alter ecosystem properties (productivity, stability, and resistance to invasion)^18^, it was recently reported that the change in species dominance would affect ecosystem processes prior to loss of species^48^,^49^,^50^. Therefore, temporal dietary changes may induce uneven abundance patterns of the gut microbiota, which may be related to short-term digestive disorders, whereas long-term dietary habits would influence gut microbiota in both species evenness and richness, as well as taxonomic composition^52^,^53^.

In the present study, there were distinct bacterial assemblages in terms of species composition, diversity, and SADs among samples from longitudinal and radial parts of the gut. Our results indicated a consistent dynamic pattern of gut microbiota in response to habitat heterogeneity, even though detailed species members could vary substantially across individuals. Considering community structure as a consequence of habitat adaptation, we inferred that concepts of species diversity and SADs derived from macro-organisms could be applied to micro-organisms and associated theory would provide underlying principles and mechanisms for interpreting the assembly patterns of microbial communities. As local environmental conditions as well as bacterial communities vary across gut sites, future research of gut microbiota in combination with measurements of resource substrates and metabolites would extract knowledge regarding how microbes interact with their environments to influence gut ecosystem functioning. In addition, considering that it is difficult to precisely assess and compare species richness across communities^51^, and even more importantly, because not only low richness but also low evenness can decrease ecosystem functioning^18^,^19^,^48^,^49^,^50^, we suggest that the evenness and the top rank species dominance might be good indicators for detection of environmental changes in the gut ecosystem.

## Methods

### Animals and gut samples

Two white-faced flying squirrels (*Petaurista alborufus lena*; namely FS1 and FS2) from the mountains of Taiwan, were used for this study. A wild animal collecting permit (No. 0990007029) was approved by Yushan National Park Headquarters. All experiments were performed in accordance with the Wildlife Conservation Act (http://law.moj.gov.tw/Eng/LawClass/LawAll.aspx?PCode=M0120001). For each flying squirrel, the food bolus (F) and mucus layer (M) from the proximal small intestine (S), cecum (C) and distal large intestine (L) were separated and preserved in the RNAlater solution (Ambion, Austin, TX, USA) for later processing. To investigate spatial heterogeneity of gut microbiota, 16S rRNA gene clone libraries from six distinct gut sites (SF, SM, CF, CM, LF and LM) were constructed and ~500 clones of each library were picked and sequenced. Details of DNA extraction, 16S rRNA amplification, cloning, and sequencing are provided (Supplementary Methods).

### Sequence analyses

The 16S rRNA gene sequences were trimmed and edited using the Sequencher program (Gene Code Corporation, Ann Arbor, MI, USA). After elimination of short, low-quality and possible chimeric sequences (verified by Bellerophon server^54^ and Chimera Slayer^55^), a total of 4768 sequences (>700 bp) were obtained. Each community contained roughly 400 sequences (total abundance). All sequences were clustered into OTUs (Operational Taxonomic Units) based on 97% sequence identity threshold, and abundances of each OTU (the number of belonged sequences) across distinct communities were calculated, using UCLUST method^56^ in the QIIME pipeline^57^. In addition, taxonomic information (from phylum to family) of each OTU was assigned using the RDP classifier^58^ in the QIIME pipeline^57^. The nucleotide sequence data reported are available in the GenBank databases under the accession numbers: JQ335999–JQ336958 and KC802244–KC806051.

### Community characteristics

Overall similarities and differences among communities were shown in ordination diagrams using PCoA (principal coordinates analysis) based on the weighted UniFrac metric^59^. Compared to short reads from next generation sequencing (454 pyrosequencing and Illumina technology), our sequences based on traditional Sanger sequencing were long enough to get reliable taxonomic annotations, yet still many OTUs were assigned to uncultured groups, especially below family level (as plentiful sequences in databases were directly from environmental samples without species isolates). Therefore, in this study, to quantify and compare species diversity across communities, “species” would refer to OTUs based on 16S rRNA gene sequence similarity as mentioned above (97% is the current criteria of the prokaryotic species definition). Species richness for each community was determined by the number of observed OTUs and potential species richness was estimated by Chao1 nonparametric estimator^60^. In addition, Shannon's diversity and evenness indices^16^ were calculated. Moreover, rarefaction curves of observed and Chao1 estimated species richness were plotted using the QIIME pipeline^57^. To reveal spatial heterogeneity of gut microbiota, each species was traced to determine its occurrence, and Venn diagrams were generated to represent the percentage (%) of overlapping species. Furthermore, species abundance distributions (SADs)^17^ annotated with phylum-level taxonomy were used to represent commonness and rarity of species. First, SADs of all bacterial communities were plotted in rank order from the most abundant species to the least abundant species (using natural log-transforming abundances). Second, to quantify the shape of the SADs, the rank was further natural-log-transformed to examine the degree of power-law decay (linear fitting) in a log-log plot. In addition, the phylum for each species was marked in different colors to visually appreciate taxonomic patterns among various gut sites.

To discuss relationships among various components of species diversity, Pearson product-moment correlation analysis was used to evaluate the correlation (r) between a pair of variables. To reveal how community structure changed with the shift of species abundances, in addition to Shannon's diversity and evenness indices, top rank species dominance (defined as the abundance proportion (%) of the most dominant species in a given community) was examined. To provide additional insights about community structure of mammalian gut microbiota, we also analyzed species diversity of bacterial communities from fecal samples of two published data sets^14^,^15^. Ley et al. study^14^ provided 17,760 sequences (NCBI Accession Number: EU458114–475873) from 85 samples based on Sanger sequencing. Muegge et al. study^15^ provided 149,675 sequences (NCBI Sequence Read Archive: SRA030940) from 39 samples based on 454 pyrosequencing. These two data sets including sufficient samples from diverse mammals allowed us to detect an overview pattern about dynamic changes of bacterial communities in gut ecosystems. Moreover, as these two independent data sets using distinct sequencing methods to generate bacterial communities, we would have chance to detect whether the sequence depth inferences the pattern we detected.

## Supplementary Material

Supplementary Information Supplementary Information

## Figures and Tables

**Table 1:** Characteristics of 12 bacterial communities from 6 gut sites (SF, CF, LF, SM, CM, and LM) of 2 flying squirrels (FS1 and FS2)

	FS1SF^a^	FS1CF	FS1LF	FS1SM	FS1CM	FS1LM	FS2SF	FS2CF	FS2LF	FS2SM	FS2CM	FS2LM
Total abundance b	397	528	389	348	382	425	365	443	435	284	368	404
Observed richness^c^	88	184	149	157	115	65	98	172	140	137	128	64
Estimated richness^d^	198	290	244	282	214	135	228	321	226	334	256	130
Shannon's diversity	2.39	4.52	4.37	4.74	3.98	2.48	2.92	4.76	4.31	4.57	4.33	2.31
Shannon's evenness	0.53	0.87	0.87	0.94	0.84	0.59	0.64	0.92	0.87	0.93	0.89	0.56

^a^See Figure 1 for explanations of community labels.

^b^Total abundance is the number of total sequences from each 16S rRNA gene clone library.

^c^Observed richness is the number of total OTUs determined by 97% sequence similarity.

^d^Estimated richness is represented by nonparametric Chao1 estimator.

**Table 2:** Species richness and mean species abundance of various bacterial phyla across 12 bacterial communities from 6 gut sites (SF, CF, LF, SM, CM, and LM) of 2 flying squirrels (FS1 and FS2)

Bacterial phylum	FS1SF^a^		FS1CF		FS1LF		FS1SM		FS1CM		FS1LM		FS2SF		FS2CF		FS2LF		FS2SM		FS2CM		FS2LM	
*Firmicutes*	81^b^	(2)^c^	173	(3)	138	(3)	145	(2)	108	(3)	39	(3)	85	(2)	155	(3)	126	(2)	125	(2)	118	(3)	54	(1)
*Proteobacteria*	3	(77)	2	(2)	3	(2)	3	(1)	-		10	(4)	9	(18)	4	(2)	4	(13)	7	(3)	4	(1)	2	(68)
*Actinobacteria*	4	(1)	8	(2)	7	(2)	7	(2)	5	(5)	13	(21)	1	(1)	9	(3)	7	(7)	2	(2)	5	(2)	7	(27)
*Deinococcus-Thermus*	-		-		-		1	(1)	-		1	(3)	1	(2)	-		-		1	(1)	-		-	
*Verrucomicrobia*	-		-		1	(2)	1	(1)	1	(7)	1	(9)	1	(2)	1	(14)	1	(31)	1	(2)	1	(11)	1	(2)
Unclassified	-		1	(1)	-		-		1	(1)	1	(1)	1	(1)	3	(1)	2	(2)	1	(3)	-		-	

^a^See Figure 1 for explanations of community labels.

^b^The number represents species richness (total OTU number belonging to the particular phylum).

^c^The number in parentheses represents mean species abundance (average sequence number per OTU).
