# Supplementary material for: Spatial heterogeneity of gut microbiota reveals multiple bacterial communities with distinct characteristics
Source: Sci Rep. 2014 Aug 26;4:6185. doi: 10.1038/srep06185 (PMC5385803; doi:10.1038/srep06185)

Supplementary Information for

"Spatial heterogeneity of gut microbiota reveals multiple bacterial communities with distinct characteristics"

Hsiao-Pei Lu[1,#], Yung-Chih Lai[1], Shiao-Wei Huang[1], Huang-Chi Chen[1], Chih-hao Hsieh[2,3,*], and Hon-Tsen Yu[1,4,*]

[1]Department of Life Science, National Taiwan University, Taipei, Taiwan, ROC 10617

[2]Institute of Oceanography, National Taiwan University, Taipei, Taiwan, ROC 10617

[3]Institute of Ecology and Evolutionary Biology, National Taiwan University, Taipei, Taiwan, ROC 10617

[4]Genome and Systems Biology Degree Program, National Taiwan University, Taipei, Taiwan, ROC 10617

[*]Corresponding author: chsieh@ntu.edu.tw

[*]Corresponding author: ayu@ntu.edu.tw

#Current address: Institute of Oceanography, National Taiwan University, Taipei, Taiwan, ROC 10617

Running head: Spatial heterogeneity of gut microbiota

Supplementary Methods

DNA extraction

Each sample was centrifuged (14,000 x g for 5 min) to remove the RNAlater solution and then re-suspended in the PBS buffer. After the PBS buffer was removed, ~0.5 g sample was homogenized in a Eppendorf tube with 300 µl lysozyme buffer (25 mM tris-HCl, 10 mM EDTA, 50 mM glucose, pH 8) containing 50 µg lysozyme (20 x 10^3 units/mg), followed by incubation at 37 °C for 2 h. Thereafter, 100 µg proteinase K (60 units/mg), 100 µg RNase A (60 units/mg) and 300 µl lysis buffer (50 mM tris-HCl, 10 mM EDTA, 1% SDS, 100 mM NaCl, pH 8) were added to each tube. Freeze-thaw cycles of incubation at -80 ℃ for 60 min and at 60 ℃ for 30 min were performed three times. The cell lysate was extracted with equal volume of phenol / chloroform / isoamyl alcohol (25:24:1), rotated to obtain a well-mixed milky solution and centrifuged (14,000 x g for 10 min) at room temperature. The top aqueous layer was placed in a new Eppendorf tube and 1 ml cold 100% ethanol (EtOH) was added. The tube was gently inverted to precipitate DNA and centrifuged (14,000 x g for 10 min) to pellet DNA. The DNA pellet was washed with 1 ml cold 70% EtOH twice and dissolved in distilled water.

16S rRNA genes amplification

A pair of primers, 27 bF (5'- AGAGTTTGATCMTGGCTCAG -3') and 1492 uR (5'-

TACGGYTACCTTGTTACGACTT -3’), were used to amplify the bacterial 16S rRNA gene fragments. The PCR reaction mixture contained 100 ng DNA, 2.0 mM MgCl2, 0.4 mM dNTP, 0.4 μM each primer, 1X Ex Taq™ buffer, and 1 U of Ex Taq™ polymerase (Takara). Thermal cycling conditions included initial denaturation at 94 °C for 2 min; followed by 25 cycles at 94 °C for 30 s, 54 °C for 30 s and 72 °C for 2 min; and final extension at 72 °C for 10 min. Amplified products of the expected size (ca. 1500 bp) were confirmed and excised from the gel, followed by DNA recovery with QIAquick Gel Extraction Kits (QIAGEN).

Cloning and Sequencing

The purified PCR products were ligated into the yT&A vector of the TA Cloning Kits (Yeastern) according to the manufacturer’s instructions. Ligated DNA was transformed into ECOS101 competent cells in the kits by heat shock at 42 °C for 45 s. The resulting clones were selected on Luria-Bertani medium with ampicillin (50 μg/ml), X-Gal (5-bromo-4-chloro-3-indolyl-β-D-galactopyranoside; 50 mg/ml), and IPTG (isopropyl-β-D-thiogalactopyranoside; 0.1 M). For each clone library, approximately 500 clones were picked and sequenced using ABI BigDye Terminator on ABI 3730xl sequencers (Applied Biosystems).

Supplementary Figures

Supplementary Figure S1. Rarefaction curves of observed species richness (a) and Chao1 estimated species richness (b). For each flying squirrel (FS1 or FS2), samples were prepared from the proximal small intestine (S), cecum (C) and distal large intestine (L), and in each section, divided into the food bolus (F) and mucus layer (M).

Supplementary Figure S2. Abundances of dominant species detected in food bolus and mucus layer of the large intestine. The dominant species was defined by those containing at least 2% of total abundances (≧8 sequences) in either LF (the food bolus of the large intestine) or LM (the mucus layer of the large intestine) in FS1 (a) and FS2 (b). Their taxonomic relationship is represented by a circular hierarchical diagram in the core with class-level taxonomy marked at the outside; the abundance of each species is represented by an external pie diagram with blue and red indicating sequences from LF and LM, respectively.

Supplementary Figure S3. Relationships among various components of species diversity of gut microbiota. The species diversity (a) and top rank species dominance (b) against richness (left-hand Y axis) and evenness (right-hand Y axis) of the 12 intestinal bacterial communities from two flying squirrels are plotted.

Supplementary Figure S4. Relationships among various components of species diversity of gut microbiota based on Sanger sequencing data sets. Species diversity (a) and top rank species dominance (b) against richness (left-hand Y axis) and evenness (right-hand Y axis) of 97 bacterial communities are plotted, based on 12 gut samples from two flying squirrels (this study) and 85 fecal samples from 56 species of mammals[1].

Supplementary Figure S5. Relationships among various components of species diversity of gut microbiota based on pyrosequencing data sets. Species diversity (a) and top rank species dominance (b) against richness (left-hand Y axis) and evenness (right-hand Y axis) of 39 bacterial communities are plotted, based on 39 fecal samples from 33 kinds of mammals[2].

1. Ley RE*, et al.* Evolution of mammals and their gut microbes. *Science* **320**, 1647-1651 (2008).

2. Muegge BD*, et al.* Diet drives convergence in gut microbiome functions across mammalian phylogeny and within humans. *Science* **332**, 970-974 (2011).

Supplementary Figure S1.

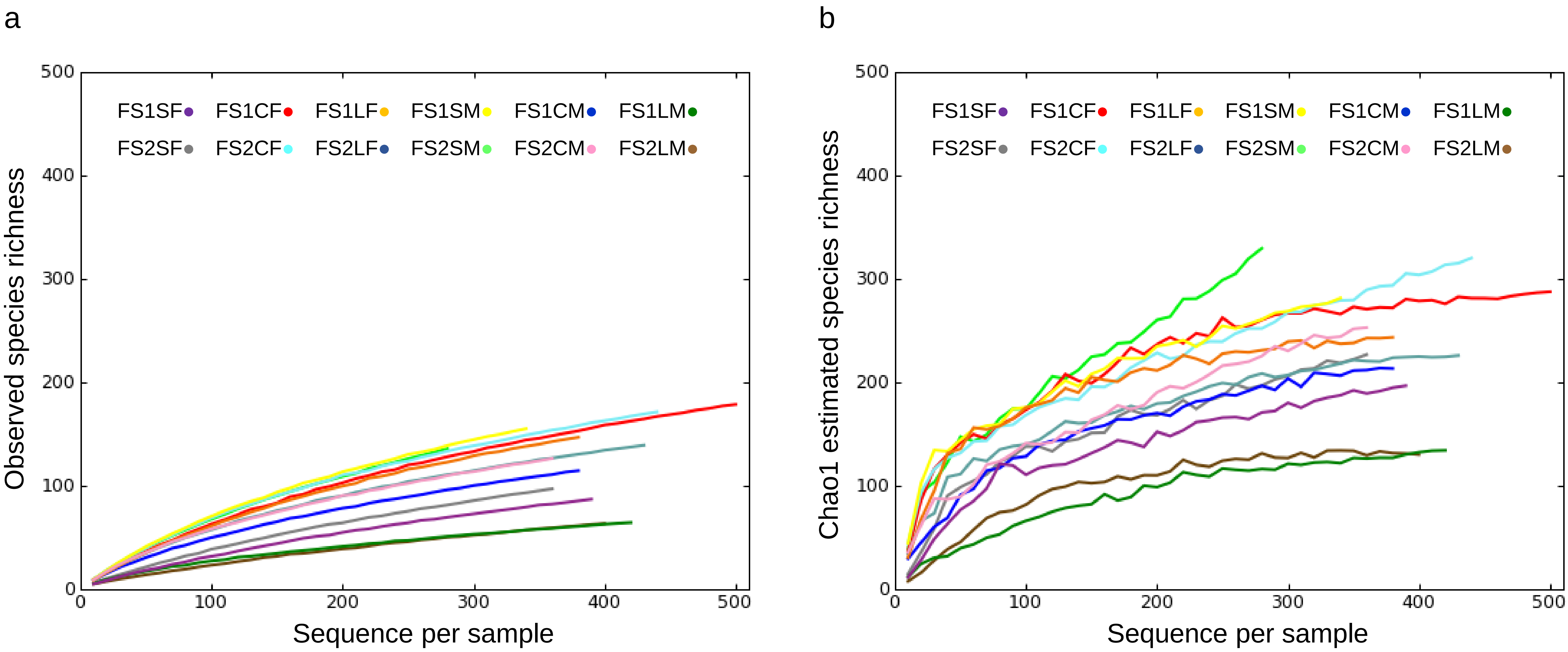

Supplementary Figure S2.

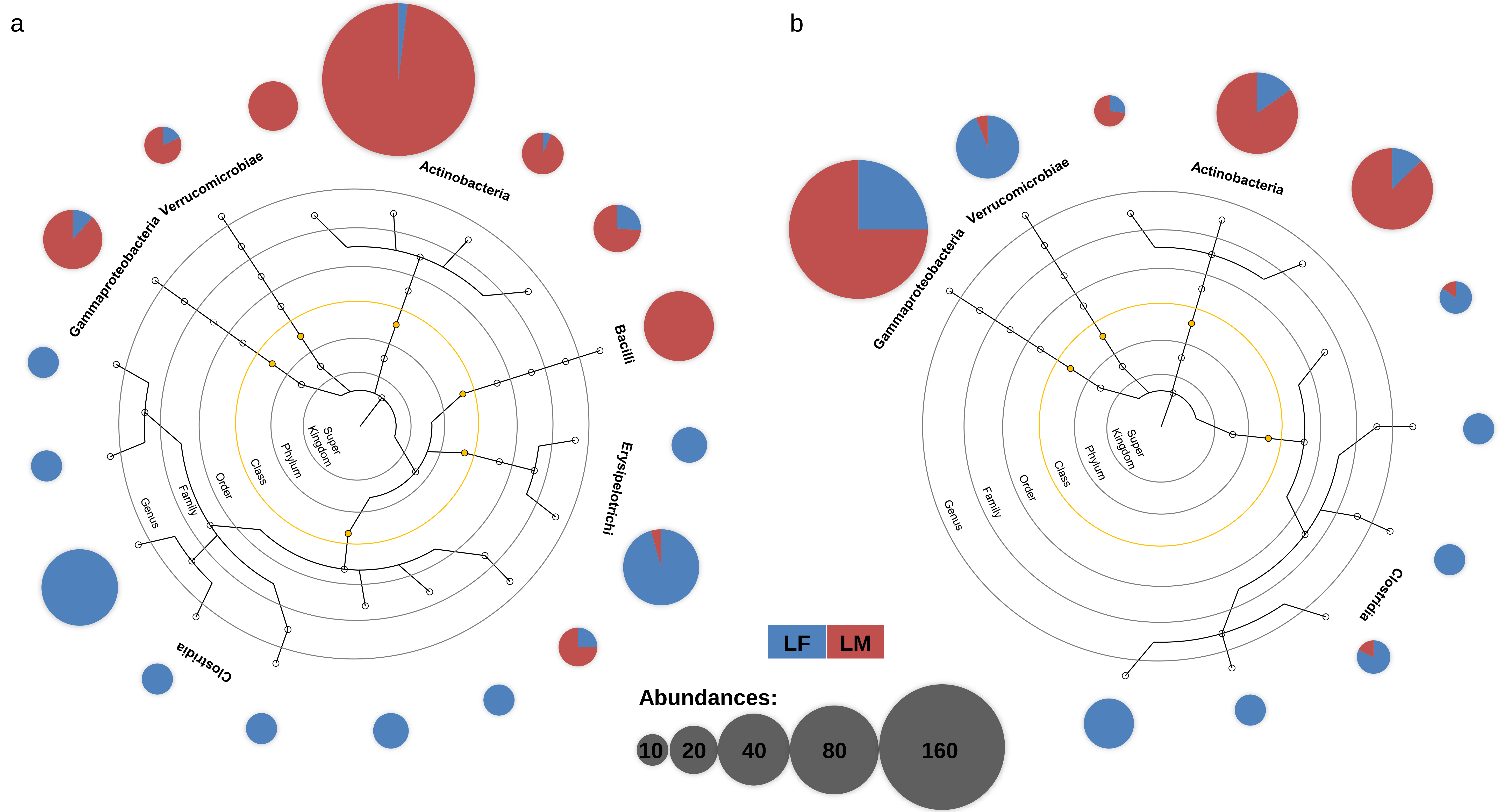

Supplementary Figure S3.

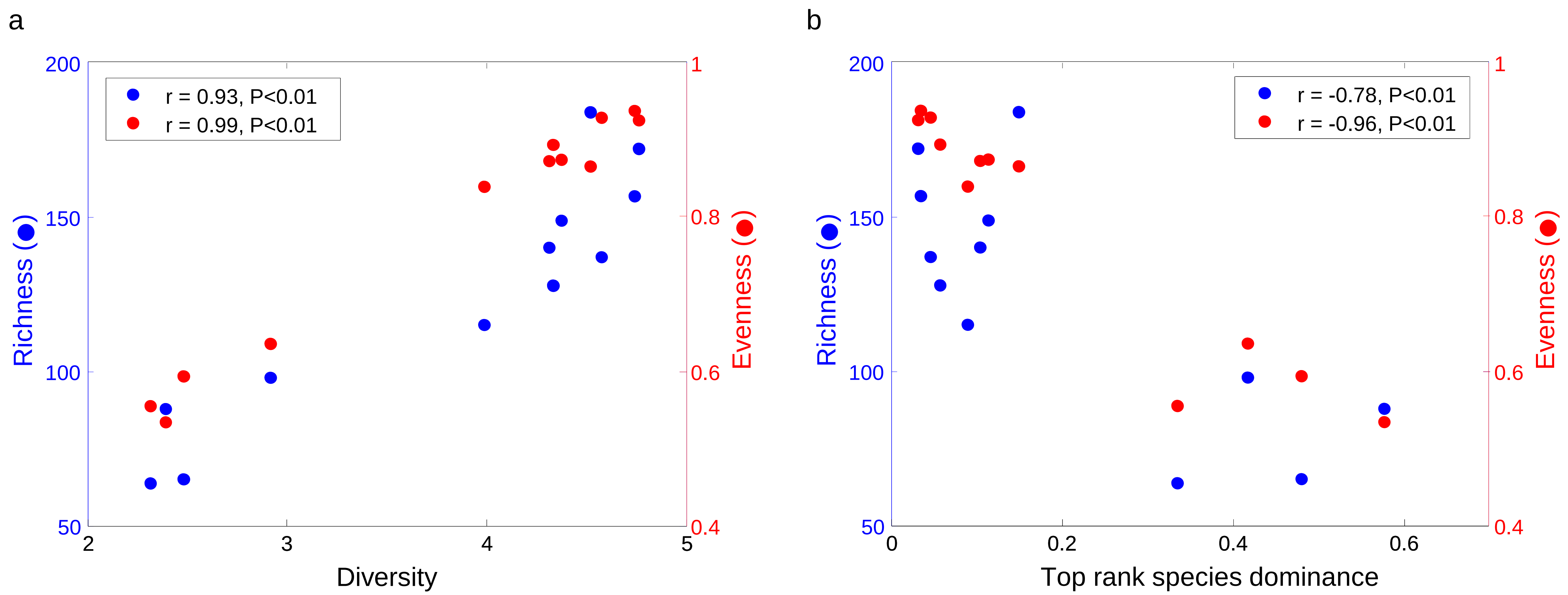

Supplementary Figure S4.

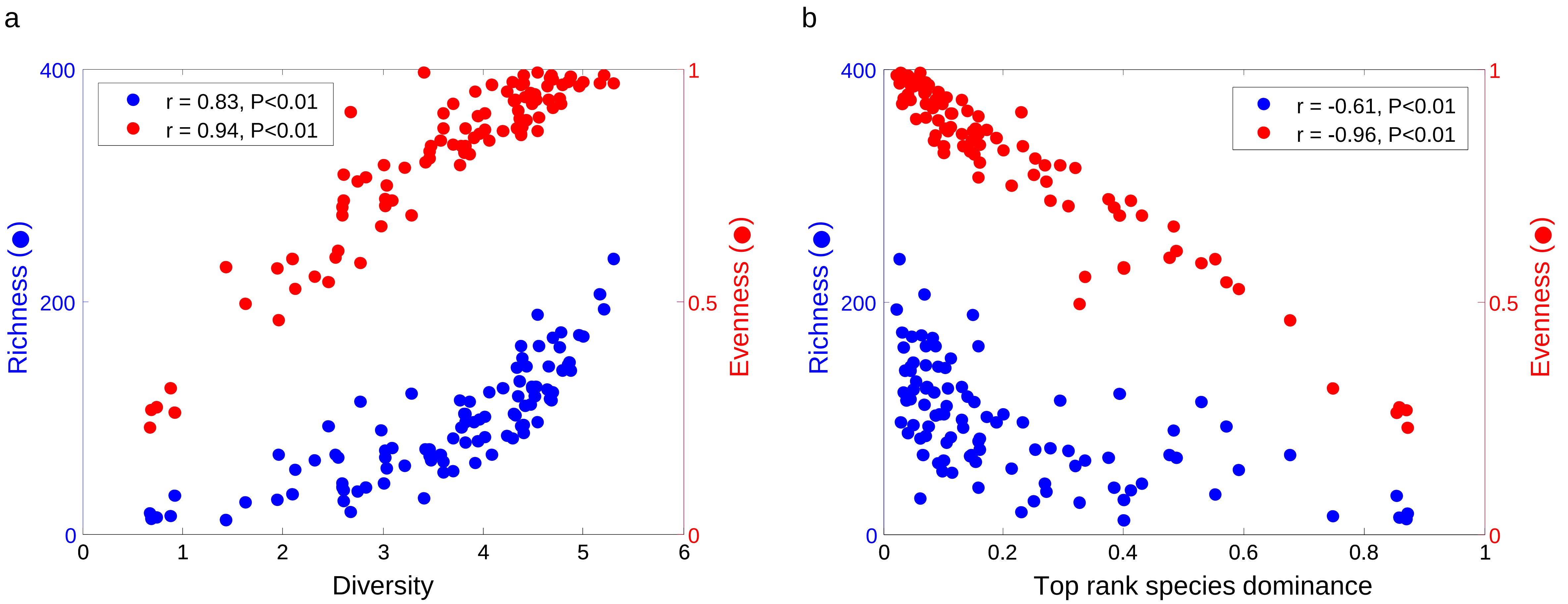

Supplementary Figure S5.

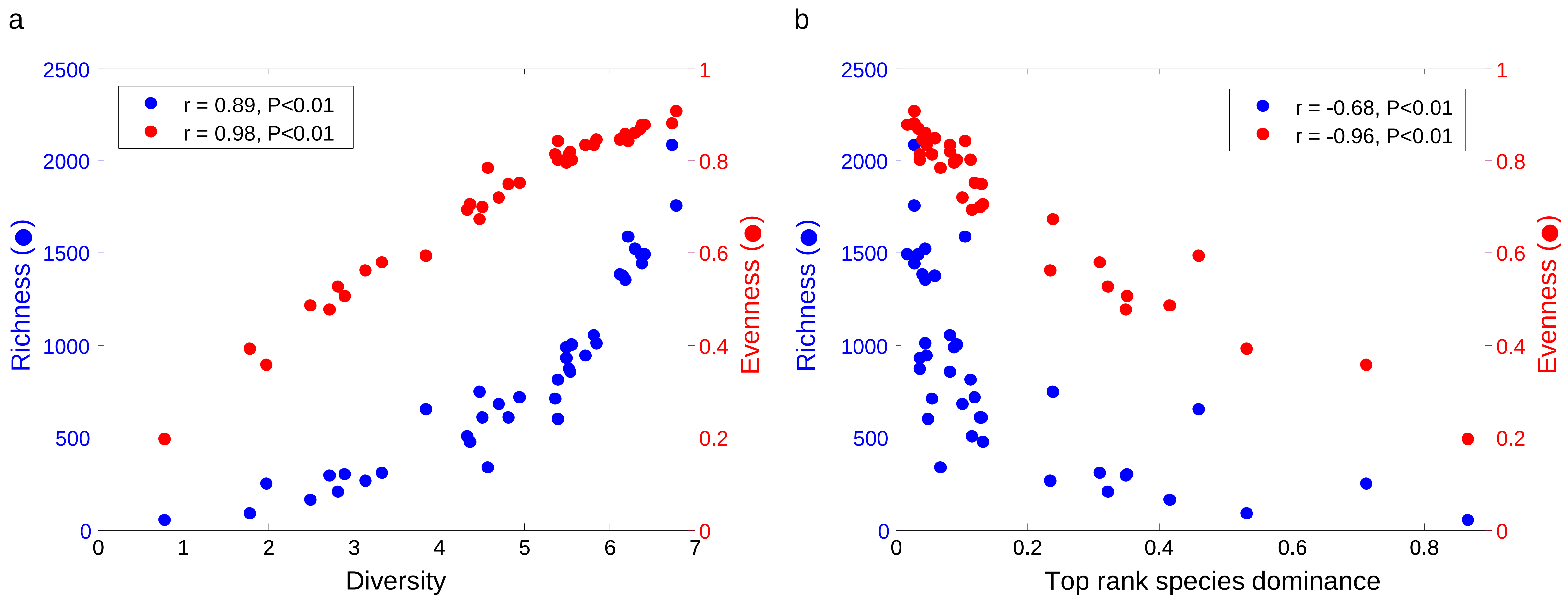

Supplement: Supplementary Information [file srep06185-s1.pdf]
